# Contribution of Protected Area Networks to Achieving Global Biodiversity Framework Targets and Sustainable Development Goals: Evidence From Coastal Shandong, China

**DOI:** 10.1002/ece3.71900

**Published:** 2025-08-17

**Authors:** Ye Zhao, Xinyu Liu, Wenyu Zhang, Nan Liu, Lin Fan, Li Zhao

**Affiliations:** ^1^ Innovation Institute for Sustainable Maritime Architecture Research and Technology Qingdao University of Technology Qingdao P.R. China; ^2^ College of Architecture and Urban Planning Qingdao University of Technology Qingdao P.R. China; ^3^ Northwest Surveying, Planning Institute of National Forestry and Grassland Administration, Key Laboratory National Forestry Administration on Ecological Hydrology and Disaster Prevention in Arid Regions Xi'an P.R. China

**Keywords:** coastal region, ecological connectivity, effectiveness assessment, global biodiversity framework, protected area network, sustainable development goals

## Abstract

The protected area network (PAN) is a vital strategy for enhancing ecological integrity and connectivity. However, its contributions to the Kunming–Montreal Global Biodiversity Framework (GBF) 2030 Targets and Sustainable Development Goals (SDGs) remain understudied due to limited indicator assessments and regional case comparisons. This study applied circuit theory to construct and optimize a PAN in China's coastal region. First, resistance surfaces were mapped, connectivity corridors identified, and network key points determined. Second, the network structure was analyzed to classify source areas and corridors by importance and to identify potential conservation zones. Finally, the PAN's contributions to GBF Targets 2 and 3, SDG 15 indicators, and related synergistic benefits were quantified using coverage‐based performance metrics and spatial analysis. The results show that the PAN raises protected‐area coverage to 33.3%, meeting the GBF 30 × 30 target, boosts SDG 15 terrestrial protection by 50% above the regional average, and supports other SDGs. Key connectivity points align along the coastal belt, with eight regions exhibiting both pinch points and barriers, underscoring the complexity of coastal–terrestrial ecotones. This study highlights the unique role of coastal PANs in global conservation and recommends refined assessment frameworks and collaborative planning to enhance their international applicability.

## Graphical abstract

This study evaluates how protected area networks (PANs) in China's coastal region advance both biodiversity conservation and sustainable development. By constructing and optimizing a PAN, we demonstrate its contributions to meeting the Global Biodiversity Framework (GBF) 2030 Targets and Sustainable Development Goals (SDGs). Our results show that the optimized PAN achieves 30% protection of critical ecosystems while enhancing ecological connectivity and SDG 15 outcomes.

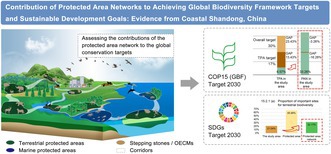

## 1 Introduction

The Kunming–Montreal Global Biodiversity Framework (GBF) and the United Nations Sustainable Development Goals (SDGs) together articulate the world's roadmap for biodiversity conservation under the 2030 Agenda. Adopted in 2015, the SDGs comprise 17 goals and 169 indicators designed to drive sustainable development across social, economic, and environmental dimensions; Goal 15 (“Life on Land”) specifically addresses the protection, restoration, and sustainable use of terrestrial ecosystems, with indicators such as 15.1.1 (forest area as a proportion of total land area) quantifying forest extent as a proxy for ecosystem integrity, while 15.2.1 (progress toward sustainable forest management) measures progress toward degraded land restoration—both critical for directly measuring area‐based conservation outcomes (Maxwell et al. 2020; UN 2022). Concurrently, the GBF sets forth 23 action‐oriented targets for 2030—including the restoration, protection, and effective management of at least 30% of terrestrial, inland water, coastal, and marine areas—to ensure ecological integrity and connectivity necessary for species survival and the provision of ecosystem services (ESs) (CBD 2022). Protected areas (PAs) are widely recognized as the cornerstone of these global targets, supporting efforts to curb biodiversity loss and sustain ecosystem functions (IPBES 2019; Watson et al. 2014). However, significant challenges remain, with only 17% of land covered by PAs by 2020, and just 7.74% of the oceans covered by MPAs—still below the GBF's 30 × 30 target (UNEP‐WCMC and IUCN 2021). This gap underscores the need to enhance PA connectivity and effectiveness (Brennan et al. 2022; Liczner et al. 2024; Nicholson et al. 2021), particularly in addressing anthropogenic pressures on ecosystem integrity and species diversity (Eckert et al. 2023).

Protected area networks (PANs) have emerged as a crucial strategy for addressing protection gaps by integrating fragmented PAs into cohesive ecological corridors. This approach enhances species migration and supports essential ecological processes (Saura et al. 2018). Over the past two decades, advancements in modeling tools—such as graph theory (Urban and Keitt 2001), least‐cost modeling (Adriaensen et al. 2003; Beier et al. 2008), circuit theory (McRae et al. 2008), and individual‐based modeling (Green et al. 2015)—have significantly enhanced the design and implementation of PANs (Keeley et al. 2021). These methodologies have proven effective in promoting species conservation (Diniz et al. 2020, 2022), supporting ecosystem restoration (Zhang et al. 2020), and enhancing ecosystem connectivity to adapt to climate change (Xu et al. 2024). Despite these advancements, challenges remain in evaluating PAN contributions to the GBF 2030 targets and SDGs. Previous studies have applied spatial panel measurements and clustering methods to assess biodiversity indicators in China (Zhang et al. 2022), used theoretical and bibliometric analyses to explore ES contributions to the SDGs (Xu and Peng 2024; Zhao et al. 2025), and examined the role of landscape patterns and ES in achieving regional SDGs (Liu et al. 2023; Zhu et al. 2024). However, the specific contributions of PANs to the realization of these global conservation goals remain poorly understood.

Recognizing PANs as a vital conservation strategy, this study argues for the construction of a regional PAN that enhances the connectivity and integrity of PAs while clarifying its contributions to GBF 2030 and SDG targets through indicator analysis and policy comparisons (Deleglise et al. 2024). Drawing on prior research that integrates landscape heterogeneity without relying on species‐specific data (Krosby et al. 2015; Liang et al. 2018), we employ least‐cost path and circuit theory modeling to construct and optimize a PAN. Quantitative evaluation methods and correlation analysis are then applied to assess the contributions of PANs to global conservation goals. This integrated approach provides a comprehensive and scalable framework for PAN assessment and implementation, offering actionable insights for conservation planning.

Coastal regions represent a dynamic interface between terrestrial and marine ecosystems, characterized by rich and unique biodiversity. However, these areas are increasingly vulnerable to the impacts of climate change, geological activity, and anthropogenic disturbances (Ferro‐Azcona et al. 2019). China's coastal regions feature various types of PAs, including terrestrial nature reserves, forest parks, geological parks, marine parks, and wetland parks, which are legally governed by instruments such as the Nature Reserve Regulation (2017) and the Ecological Redline Policy (2024). These policies mandate systematic protection of critical ecological areas while guiding the transition from “isolated protection” to holistic management. Shandong Province, the second most populous coastal province in eastern China, has an extensive coastline, and intense human activities create complex, fragile ecosystems. MPAs in Shandong cover 9419 km^2^ (5.5% of the provincial marine area), exceeding the national MPA coverage rate of 4.1%. In contrast, terrestrial protected areas (TPAs) span only 7563 km^2^ (4.8% of land area), significantly below the national TPA average of 18% and the GBF's 30 × 30 target for 2030 (Department of Natural Resources of Shandong Province 2023; Li and Pimm 2020). Under the policy‐driven shift toward “systematic and holistic protection” (Xu and Pan 2019), Shandong's PAs face urgent challenges in enhancing connectivity and aligning with national conservation mandates. This highlights the need to coordinate PAs with coastal landscapes through PAN development.

In this study, the coastal region of Shandong Province was selected as a case study, with two primary research objectives: (1) to construct a coastal PAN that integrates ecological suitability and avoids anthropogenic disturbance, optimizing its structure to enhance the efficiency of existing PAs and improve connectivity, and (2) to evaluate the extent to which the constructed PAN contributes to the achievement of relevant GBF 2030 targets and SDGs. This work introduces a novel approach by combining both TPAs and MPAs in a spatially integrated model and applying a multi‐layer connectivity analysis to assess PAN effectiveness. Through quantitative methods, this study identifies the critical contributions of PANs to global conservation targets, offering practical insights that could inform future conservation planning and policy development, particularly in densely populated and highly impacted coastal regions.

## 2 Materials and Methods

### 2.1 Study Area

**FIGURE 1:**
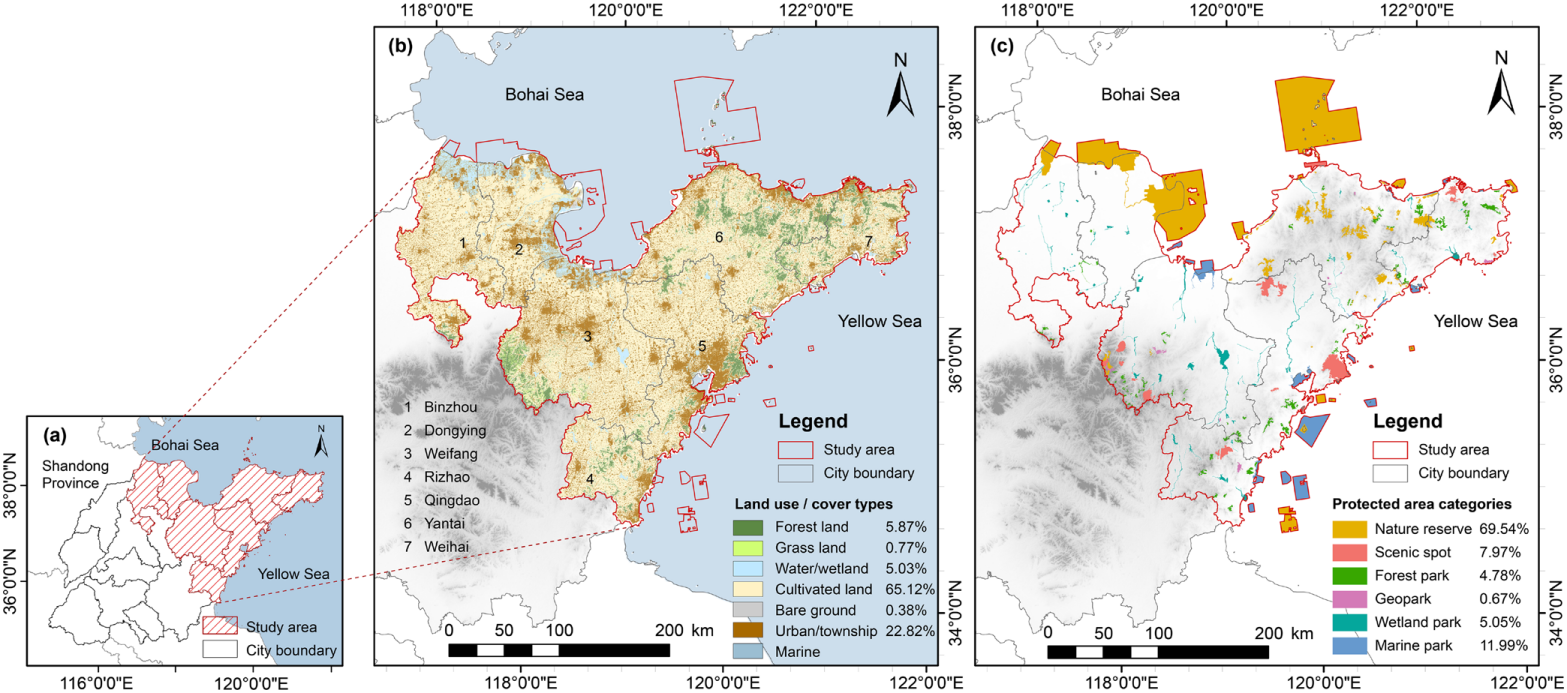
Study area location and scope. (a) Study area in Shandong Province. (b) Administrative map and land use/cover types. (c) Protected area (PA) categories.

The study area is located along the eastern coast of the Shandong Peninsula, China (35°07′–38°40′ E, 117°27′–122°72′ N), bordering the Bohai Sea and the Yellow Sea, and includes seven coastal cities (from west to east: Binzhou, Dongying, Weifang, Rizhao, Qingdao, Yantai, and Weihai) (Figure 1). The total area of this region is 78,325.32 km^2^, accounting for 55.09% of the total terrestrial and marine area of Shandong Province, with a terrestrial area of 69,921.32 km^2^ and a marine area of 8404.00 km^2^. In this area, there are 171 PAs (266 independent patches) comprising nature reserves (*n* = 29), scenic spots (*n* = 17), forest parks (*n* = 38), geological parks (*n* = 8), wetland parks (*n* = 60), and marine parks (*n* = 19), covering a total of 12,997.74 km^2^ (see Appendix A for more details). Terrestrial and inland water PAs occupied 6.57% of the total terrestrial area in the study area; MPAs covered 19.44% of the jurisdictional marine territory across the seven cities. The main wildlife species protected in the study area include migratory birds, small and medium‐sized mammals, and aquatic organisms, with a decreasing trend in species richness from east to west. The area comprises several terrestrial ecosystems, including forests, scrublands, meadows, and wetlands, as well as four marine ecosystems: coastal zones, islands, estuaries, and offshore (Department of Ecology and Environment of Shandong Provincial 2021).

### 2.2 Datasets

Seven spatial datasets were used in this study: four raster and three vector (Table 1). All environmental data were standardized into a projected coordinate system with a cell size of 30 m × 30 m in raster format.

**TABLE 1:** The datasets adopted in our study.

Dataset name	Type	Source	Year	Resolution	Application in the study
Protected area boundaries	Vector	Department of Natural Resources of Shandong Province, and the data are as of April 2023. http://dnr.shandong.gov.cn/zwgk_324/gs/202304/t20230410_4287411.html	2023	N/A	Corridor modeling
Biodiversity conservation priority area boundaries	Vector	Strategy and Action Plan for Biodiversity Conservation in Shandong Province (2021–2030) http://xxgk.sdein.gov.cn/zfwj/lhf/202106/t20210603_3620268.html	2023	N/A	Evaluating the protection scope of PAN
China Land Cover Dataset (CLCD)	Raster	The 30 m annual land cover datasets and its dynamics in China from 1985 to 2022 https://zenodo.org/record/8176941	2022	30 m	Resistance mapping
Digital elevation model (DEM)	Raster	SRTM (Shuttle Radar Topography Mission, SRTM) https://srtm.csi.cgiar.org/srtmdata/	2022	30 m	Resistance mapping
Road network and water distribution data	Vector	OpenStreetMap www.openstreetmap.org	2023	N/A	Resistance mapping
Global NPP‐VIIRS‐like nighttime light data	Raster	An extended time‐series (2000–2018) of global NPP‐VIIRS‐like nighttime light data. https://doi.org/10.7910/DVN/YGIVCD	2022	500 m	Resistance mapping

### 2.3 Methodological Framework

**FIGURE 2:**
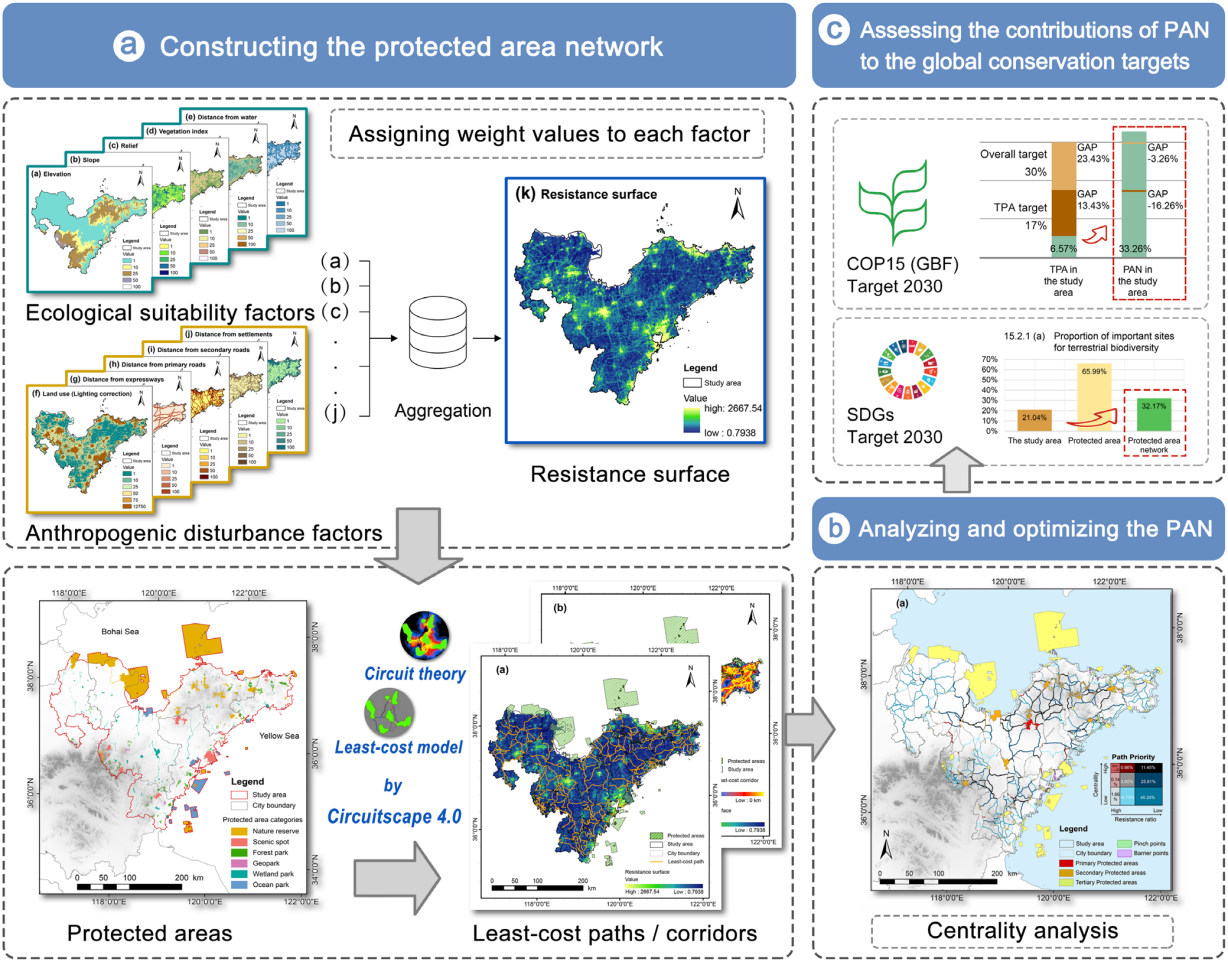
Method framework for PAN construction, optimization, and evaluation.

To construct a PAN in the coastal region and evaluate its contributions to the GBF and SDGs, a three‐step methodological framework was applied (Figure 2). First, the PAN was constructed through the steps of resistance mapping, connectivity corridors identification, and network key point identification. Second, the network structure was analyzed and optimized to classify the importance of source areas and corridors and to identify potential conservation zones. Finally, the contributions of the PAN to the GBF 2030 targets and SDGs were assessed, focusing on the achievement of GBF Targets 2 and 3, SDG 15 indicators, and other synergistic benefits.

### 2.4 PAN Construction

#### 2.4.1 Mapping the Resistance Surface

A composite resistance surface was constructed based on both ecological suitability and anthropogenic disturbance gradients (Cameron et al. 2022). First, the selection of resistance factors was guided by their significant impact on the connectivity of PAs, integrating both ecological suitability and human influence (Poor et al. 2012; Williamson et al. 2023). Natural factors included elevation (E1), slope (E2), relief (E3), Normalized Difference Vegetation Index (NDVI, E4), and distance to water bodies (E5). These factors are commonly used to represent habitat quality and ecological gradients that affect species movement and ecological processes. Anthropogenic factors included land‐use type (A1), distance from expressways (A2.1), distance from primary roads (A2.2), distance from secondary roads (A2.3), and distance from settlements (A3). These factors represent various intensities of anthropogenic disturbance and are widely recognized as key determinants in landscape resistance modeling (Zhao, Qian, et al. 2024). Second, we created a scoring quantitative questionnaire and invited 10 experts in the field of PAs (2 from the National Forestry and Grassland Administration, 3 university professors, 2 wildlife conservation enthusiasts, and 3 local residents) to score each factor according to the degree of its influence on the connectivity of the PAs (Appendix B). The results of the scores were summarized and converted into relative importance weights for the factors through a structured assessment process, with the greater the influence of the factor, the higher the weight. The Analytic Hierarchy Process (AHP) was applied using *yaahp* software to determine the relative importance and priority weights of each factor (Hurley et al. 2009). Third, following previous studies, factor values were reclassified into five resistance levels (1, 10, 25, 50, 100) to represent varying degrees of movement resistance (Rayfield et al. 2010). A value of 1 indicated unimpeded movement, while 100 represented a complete barrier (Huang et al. 2021). Each factor's resistance levels were then used to create individual resistance surfaces. Finally, the single‐factor surfaces were aggregated using the Raster Calculator tool in ArcGIS 10.8, weighted by their respective scores, to generate the composite resistance surface.

#### 2.4.2 Identification of Linkage Corridors

After determining PAs as sources and integrating resistance surfaces, this study utilized the Linkage Pathways tool in the Linkage Mapper toolbox to generate linear corridors of least‐cost pathways as well as ecological flow corridors with width information (Liang et al. 2018). Least‐cost pathways were derived from the sum of the cost‐weighted distance rasters calculated for each pair of connected core areas and normalized to the least‐cost corridors by subtracting the least‐cost pathway distances from the original corridors. These are the least‐cost linear paths for connectivity between PA patches and are considered the best paths for species migration and ecological flow in heterogeneous landscapes (Thiele et al. 2018). Circuit theory models connectivity corridors by simulating the movement and dispersal of individuals or genes across a landscape using a random walk of charges in a circuit to calculate current density (McRae et al. 2008). This multipath approach predicts the relative accuracy of all possible dispersal pathways in heterogeneous landscapes (Fletcher Jr. et al. 2023) and identifies multiple potential pathways without relying on species‐specific migration data, thereby assessing broad connectivity between known destinations (Kumar and Cushman 2022). Iterative analysis revealed that setting the cumulative resistance threshold at 10 K produced corridor widths ranging from 100 to 3000 m, meeting the migration and dispersal needs of most medium‐to‐small terrestrial animals (Merkle et al. 2023).

#### 2.4.3 Identification of Key Points That Affect the Connectivity of PAs

The key ecological points (pinch and barrier points) in the corridors were identified using Circuitscape 4.0 (McRae et al. 2017). Pinch points are areas of high current density in the PAN where species migration is likely to occur or where there are no alternative routes (McRae et al. 2012). The deterioration or loss of biodiversity in these areas is highly likely to disrupt connectivity between PAs. Therefore, prioritizing the protection of these areas is critical when constructing a PAN. We used the Pinchpoint Mapper tool in the Linkage Mapper Toolbox to invoke the Circuitscape program, using the “all‐to‐one” mode and setting a resistance cost‐weighted distance of 10 km as the “width” for current density analysis (Saura et al. 2019). In ArcGIS 10.8, the current density values were classified into five classes using the natural breaks (Jenks) method. This method minimizes the class mean squared deviations while maximizing the mean value deviation between classes, thus aggregating each value based on its similarity and is well suited for illustrating significant changes (ESRI 2023). The higher the current centrality value, the higher the area rank, and the more likely it is to be a pinch point. Patches larger than 0.1 km^2^ at the highest density level were extracted as pinch points to improve the overall network connectivity.

Barrier points are landscape features that hinder the movement of species between patches; removing or repairing them can significantly improve the connectivity between PAs (McRae et al. 2012). The Barrier mapper tool was used to calculate the improvement scores within the corridor. Using a moving window analysis with the minimum detection radius set to 100 m, the maximum detection radius set to 500 m, and a radius step value of 200 m, the cell values in the center of the search window were replaced with the least‐cost distance value between sources. Quantifying the improvement in connectivity by the least‐cost distance value, repairing, or restoring high‐value barrier points in the corridor enhances connectivity between PAs. We identified key point areas by extracting regions classified at the highest level in the five‐class natural breaks method, with an area larger than 0.05 km^2^, and pinpointed their geometric centers as barrier points. This approach helps prevent fragmentation and minimizes errors potentially caused by insufficient data accuracy.

### 2.5 Analyzing and Optimizing the PAN Structure

The PAN structural optimization framework operates at three interconnected levels: patch (PA patches), corridor (linkage pathways), and node (network key points). Centrality analysis was employed to assess the importance of links and core areas in maintaining overall network connectivity, thereby quantifying the contribution of the PAN's spatial structure to landscape connectivity. Using the Centrality Mapper tool, current centrality values were calculated across the network (Beier et al. 2011), and the natural breaks method classified PA patches and corridors into three priority levels: high (III), medium (II), and low (I) (PA patches I: 0–3427.012, II: 3427.013–3542.409, III: 3542.410–9983.385; corridors I: 0–661.356, II: 661.357–1358.600, III: 1358.601–4597.630). To prioritize PA patches and least‐cost paths for conservation or restoration, we integrated the relative resistance ratio (Barnett and Belote 2021) with a statistical analysis of the cumulative weighted distance (CWD) to actual path length ratio (McRae and Kavanagh 2011). This ratio, which quantifies the relative resistance of linkage pathways, was calculated for all least‐cost paths derived from the composite resistance surface. Using the natural breaks method, we classified these paths into high‐resistance, medium‐resistance, and low‐resistance categories, enabling targeted prioritization of ecological linkages based on their resistance characteristics. The framework also identified pinch points—critical migration corridor areas that act as stepping stones and significantly enhance the survival of long‐distance migratory species (Saura et al. 2014). Conversely, barrier points, associated with impassable natural environments or severe anthropogenic disturbances, were identified as areas requiring restoration. By integrating key factors influencing PA connectivity into the patch–corridor–node analysis, a prioritized and actionable PAN was developed. This optimized structure provides practical guidance for enhancing ecological connectivity and addressing obstacles in existing PA patterns in coastal regions.

### 2.6 Assessing the PAN Conservation Contributions

#### 2.6.1 Contributions of PAN to the GBF 2030 Targets

At least eight (1, 2, 3, 4, 8, 11, 12, and 14) of the GBF 2030 targets explicitly mention area‐based conservation measures that call for expanding the area of PAs and enhancing ecological integrity and connectivity (CBD 2022). This study evaluates the PAN contributions to achieving the GBF 2030 targets from two dimensions. First, we assess area expansion by calculating the total area of the PAN, including PAs and corridor boundaries, with a cumulative cost distance of 17 km (the average of the least‐cost distances between PA patches) and comparing the proportion of the study area to the requirement of 30% of the protection area in GBF Targets 2 and 3. Second, we assess PA connectivity enhancement by consulting the planning document for the Terrestrial Biodiversity Conservation Priority Areas (BCPAs) in Shandong Province (Department of Ecology and Environment of Shandong Province 2021) and comparing the boundaries of these priority areas with the PAN to propose an ecological pattern to enhance connectivity of PAs in the coastal region of Shandong Province.

#### 2.6.2 Contributions of PAN to the SDGs

A quantitative analysis was conducted to assess the contribution of PAN to coverage‐based indicators of the SDGs, with emphasis on SDG 15 due to its specific focus on terrestrial ecosystem conservation (UN 2022). Specifically, this study calculated the capacity of PAs, PAN, and the overall study area to meet the four representative sub‐goals of SDG 15 (15.1.1, 15.1.2, 15.3.1, and 15.4.1) (see Appendix C). Furthermore, a correlation analysis was performed on the statistical results of these six sub‐goals to demonstrate the synergies between them (Wu et al. 2022).

## 3 Results

### 3.1 PAN Construction

#### 3.1.1 AHP Results and Resistance Map

In the context of constructing the resistance surface, the AHP was employed to determine the weights of factors E1, E2, E3, E4, E5, A1, A2.1, A2.2, A2.3, and A3. As depicted in Table 2, a pairwise comparison matrix was established. This matrix reflects the relative importance of these factors through pairwise comparisons, serving as the foundation for subsequent weight calculations. Subsequently, the calculated weights are presented in Table 3, summing to 1 as required by AHP. A consistency check was then performed. The Consistency Ratio (CR) was calculated to verify the consistency of the pairwise comparisons. The result showed that the CR value was 0.0088, which is less than the acceptable threshold of 0.10. This indicates that the judgments in the pairwise comparison process were consistent, and the derived weights of the resistance surface factors are reliable. These weights can accurately reflect the relative significance of each factor in the construction of the resistance surface.

**TABLE 2:** Comparison matrix of the resistance factors.

Criteria	E1	E2	E3	E4	E5	A1	A2.1	A2.2	A2.3	A3
E1	1.000	0.957	1.032	0.985	1.049	0.405	0.463	0.447	0.440	0.430
E2	1.045	1.000	1.078	1.029	1.107	0.423	0.480	0.467	0.460	0.449
E3	0.969	0.928	1.000	0.955	1.017	0.393	0.445	0.433	0.427	0.420
E4	1.1015	0.972	1.047	1.000	1.065	0.411	0.465	0.453	0.446	0.437
E5	0.953	0.903	0.983	0.939	1.000	0.386	0.436	0.426	0.419	0.412
A1	2.468	2.364	2.545	2.433	2.591	1.000	1.142	1.102	1.086	1.071
A2.1	2.160	2.083	2.247	2.151	2.294	0.876	1.000	0.962	0.943	0.930
A2.2	2.237	2.141	2.310	2.208	2.346	0.907	1.039	1.000	0.980	0.965
A2.3	2.272	2.174	2.343	2.243	2.387	0.921	1.060	1.020	1.000	0.985
A3	2.326	2.227	2.381	2.287	2.427	0.934	1.075	1.036	1.015	1.000

**TABLE 3:** Weight of resistance factors.

Criteria	Weight
E1	0.0836
E2	0.0874
E3	0.0810
E4	0.0849
E5	0.0797
A1	0.2062
A2.1	0.0857
A2.2	0.0779
A2.3	0.0236
A3	0.1899
Total	1

**FIGURE 3:**
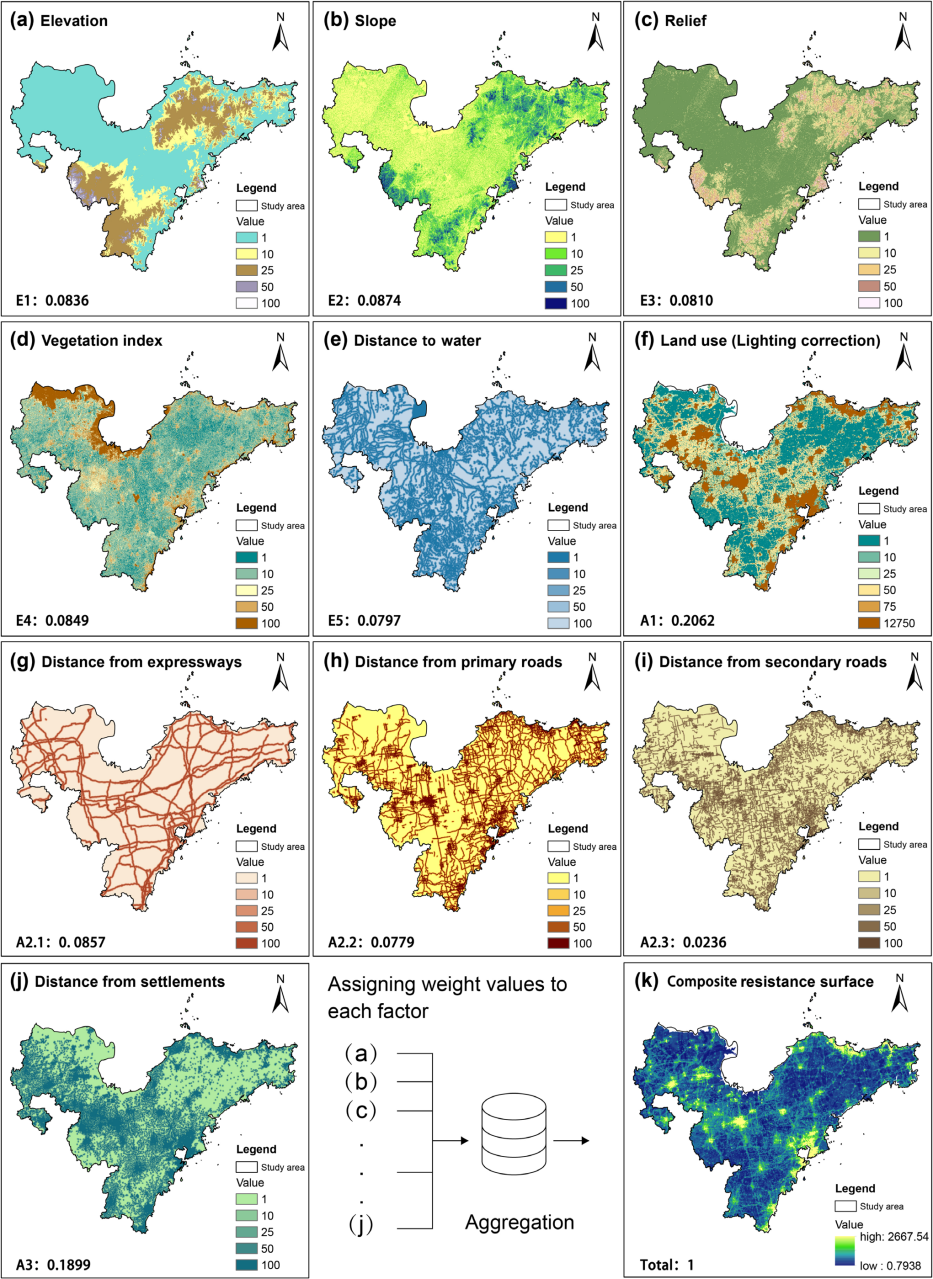
Resistance surface. The resistance factors being selected include two aspects: ecological suitability and human influence. Environmental data was scored based on expert opinions, and single‐factor resistance surfaces were reclassified and mapped accordingly. A higher resistance value indicates a greater degree to which environmental factors hinder connectivity. Ecological suitability resistance surfaces include (a) elevation, (b) slope, (c) relief, (d) Normalized Difference Vegetation Index (NDVI), and (e) distance to water bodies; anthropogenic disturbance resistance surfaces include (f) land use types, (g, h, i) distance from roads, and (j) distance from settlements. (k) Illustrate that after the reclassification of single‐factor resistance surfaces, they were superimposed according to the corresponding weights to form a composite connectivity resistance surface. The areas with high resistance values are mainly concentrated in central urban areas and along transportation routes within the study area.

Using the weighted sum overlay tool in the GIS, the five ecological suitability resistance surfaces and the five anthropogenic disturbance resistance surfaces were aggregated into a composite resistance surface based on their respective weights, with the highest resistance value reaching 2667.54 (Figure 3). Combined with the spatial distribution characteristics of high‐resistance areas (colored yellow), the following findings were obtained. (1) In terms of city development level, high‐resistance areas were mainly concentrated in central urban areas and along major transportation routes in the study area; in contrast, low‐resistance areas were associated with underdeveloped regions, including mountainous areas, forests, and remote villages. Furthermore, four of the seven coastal cities had high‐resistance areas located along the coastlines (Rizhao, Qingdao, Yantai, and Weihai), whereas the high‐resistance areas of the remaining three cities were inland (Binzhou, Dongying, and Weifang), which aligns well with the administrative centers and densely populated areas of these cities. Some areas at transportation hubs also exhibited higher resistance values, reflecting the significant interference of transportation on connectivity. (2) In terms of land‐use types, the resistance values of green spaces such as mountains, forests, and parks within urban areas were significantly lower than those of the surrounding areas, indicating that favorable natural conditions within cities reduced resistance to connectivity despite human activities. These characteristics indicate that anthropogenic disturbance is the primary factor affecting coastal connectivity, and resistance values are strongly positively correlated with population density and degree of land development.

#### 3.1.2 Least‐Cost Paths and Potential Corridors

**FIGURE A1:**
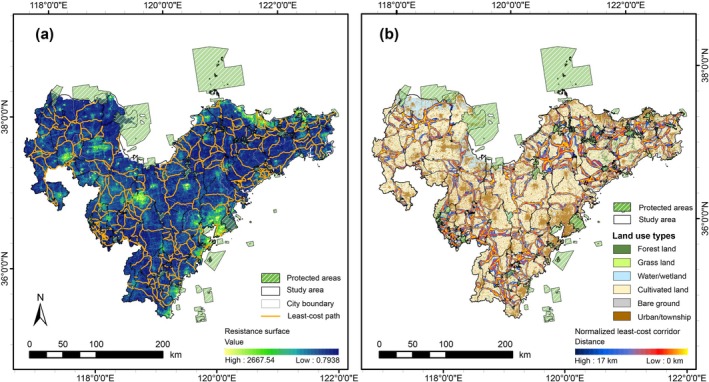
Identification of linkage corridors. (a) A total of 546 least‐cost paths were identified, which represents the most efficient linear routes between PAs. (b) Potential corridors with normalized least‐cost distance, the main alignments of the potential corridors were consistent with the least‐cost paths, both avoiding areas with high resistance values.

A total of 546 least‐cost paths were identified in the study area (Figure A1, Appendix D), covering 9415.76 km and averaging 17.24 km per path, which signified the most efficient linear routes between PAs. The longest path was 94.21 km, whereas the shortest span was only 42 m, with 344 paths falling below the average length. Additionally, the Linkage Mapper produced potential corridors based on normalized least‐cost distances, outlining all feasible connectivity pathways between the PAs (Figure A1). When the normalized least‐cost distance reached 17 km (the average of the least‐cost distances between PA patches), the land use type with the largest share within the corridor was cultivated land at 73%, followed by urban/township land at 13.04%. This result emphasizes that types of land used by humans are predominant conversion targets when designing and implementing corridors.

**FIGURE 4:**
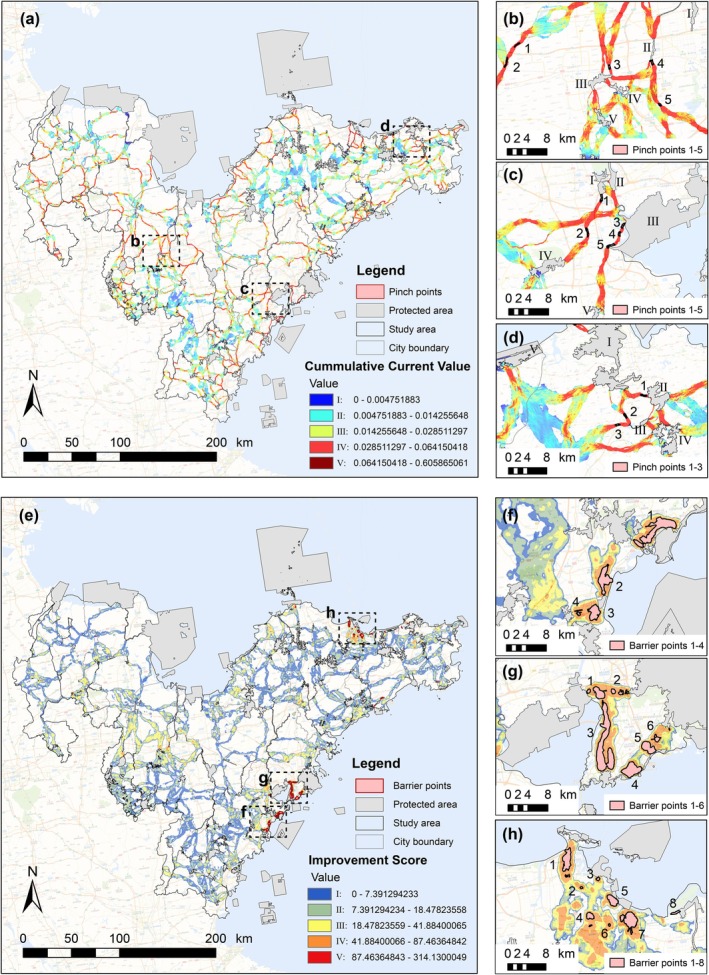
Pinch points and barrier points identification. (a) Cumulative current density map and pinch points with the highest values. (b) Features of five pinch points in the western part of the central urban study area. (c) Features of five pinch points in the urban coastal region of the southeastern study area. (d) Features of three pinch points in the urban mountain area of the northeastern study area. (e) Improvement scores map and barrier points with the highest scores. Distribution characteristics of barriers in the Qingdao coastal suburb (f), the central urban areas of Qingdao (g), and the northeast coastal region of Yantai (h).

Figure 4a shows the cumulative current flow density map within the PAN, which represents the likelihood of species movement along the least‐cost corridors. The areas with higher current densities, depicted in red, indicate an increased probability of species transit. There are 109 pinch points identified in the corridors, covering a total area of 43.46 km^2^. These pinch points represent areas of peak current within the corridor, typically averaging no more than 500 m in width, and are scattered throughout the study area, primarily in the narrowest corridor areas. The spatial types of distribution of pinch points in coastal cities were found to be of three types: central urban (Figure 4b), urban coastal (Figure 4c), and urban mountainous areas (Figure 4d).

By extracting key point areas larger than 0.05 km^2^ and pinpointing their geometric centers, 44 barrier points were identified (Figure 4e). The overlay analysis of land use types indicated that these barrier points were predominantly concentrated in densely populated coastal urban regions, particularly in the eastern and southeastern study area sectors. These barriers were primarily caused by transportation infrastructure and urban expansion. For example, Figure 4f–g illustrate the barrier distribution along the Qingdao coastline and show a clear correlation between population density and barrier density. Barrier concentrations correlate positively with population density, increasing from four locations in suburban areas (Figure 4f) to six locations in central urban zones (Figure 4g). These points are situated in the coastal region ecotone, which serves as a transitional zone between natural and artificial ecosystems. They lie on essential pathways that connect PAs within the urban environment. Figure 4h illustrates the distribution of eight barriers in Yantai Bay that act as obstacles to the connectivity between marine parks, natural reserves, and forest parks in the vicinity. Therefore, targeted improvements at these barriers in coastal regions can enhance the connectivity between terrestrial and MPAs, thereby significantly improving coastal PAN integrity.

### 3.2 PAN Optimization

**FIGURE 5:**
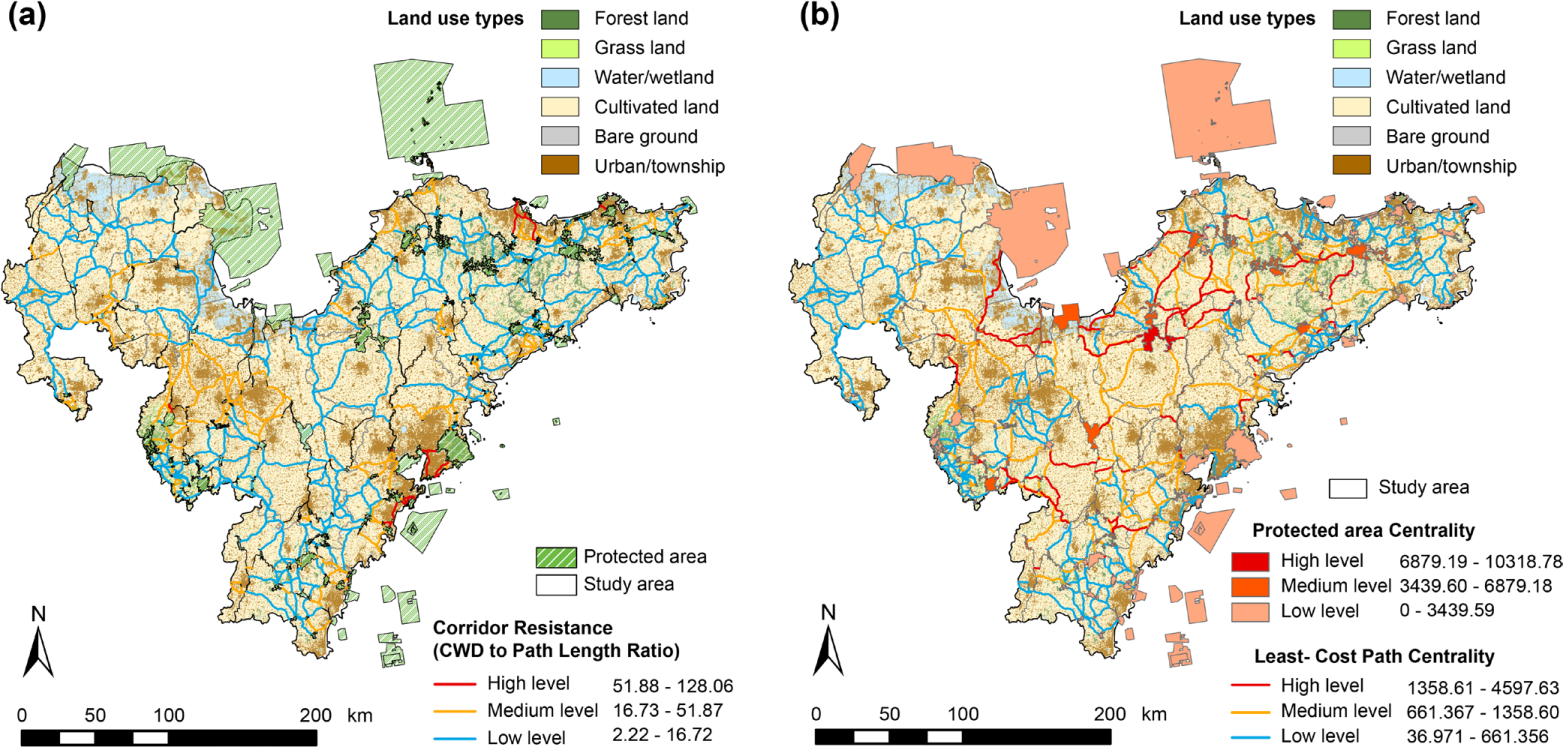
The structural analysis of the PAN. (a) The relative resistance between the linkages is reflected by the ratio between CWD and path length, and the linkages have been classified into three levels using the natural breaks method. (b) The results of the centrality analysis of the PAN; the PA patches along with the linkage pathways have been classified into three levels of centrality, distinguished by different colors.

Our statistics revealed that within the study area, there were 360 low‐resistance pathways, 166 medium‐resistance pathways, and 20 high‐resistance pathways (Figure 5a). In the PAN, both PA patches and linkage pathways were classified into three levels using the natural breaks method based on the values of the current flow centrality (Figure 5b). There were four high‐centrality PA patches (the first level), totaling 279.96 km^2^ and representing only 2.15% of the total area of PAs. Despite their limited coverage, these areas were vital to the connectivity and integrity of the PAN and should be given a conservation priority. Furthermore, there were 33 PA patches with medium centrality (the second level), covering a total area of 1239.37 km^2^, which represented 9.54% of the total area of PAs. The low‐centrality PA patches (the third level) were the most numerous, with 229 patches encompassing 11,478.40 km^2^, representing 88.31% of the total area of PAs.

**FIGURE 6:**
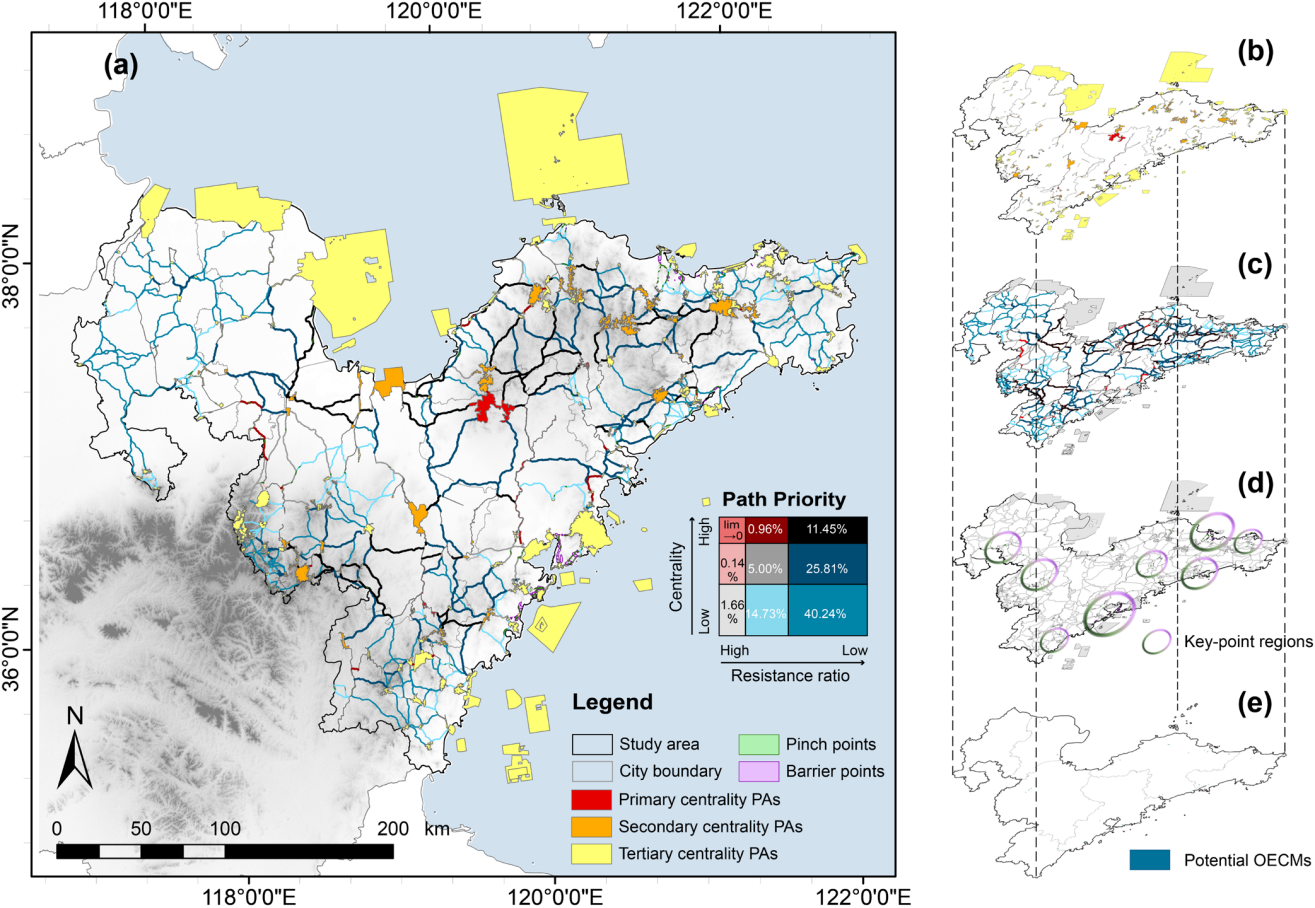
PAN optimization. (a) The PAN, composed of PA patches and linkage pathways, is visualized with varying priority levels and network key points. Grid size and percentage labels indicate the proportion of different pathway types in each dataset. (b) PA patches are categorized into three connectivity centrality levels, represented by three distinct colors. (c) Linkage pathways are classified into three priority levels—low, medium, and high—based on resistance ratio and connectivity centrality. Nine pathway types are aggregated into a bivariate agreement ensemble map, distinguished by nine colors to reflect corridor construction priorities. (d) Eight regions with pinch points and barrier points are identified. (e) Illustrates the identification results of potential other effective area‐based conservation measures (OECMs).

The PA patches, linkage corridors, and key network points were integrated to create a PAN that enhanced the existing PA pattern connectivity (Figure 6a). PA patches classified into three levels of connectivity centrality were considered the network cores (Figure 6b), with the PA of Dazeshan (red) in northern Qingdao considered the most important, as it connects multiple PA types. Utilizing the analysis of the relative resistance ratio and connectivity centrality, we aggregated nine types of pathways to distinguish corridor construction priorities and generated a bivariate agreement ensemble map distinguished by nine colors (Figure 6c), providing policymakers with multiple options for constructing connectivity corridors. Key points in the network were predominantly identified in the coastal regions of Qingdao and Yantai (Figure 6d). Additionally, eight regions contained pinch points with high connectivity probability and barrier points of high resistance, three of which were located inland and the remaining five on the coastline, indicating the prioritized importance of marine–terrestrial ecotones in conservation actions.

As the key areas for maintaining ecological flow, pinch points can serve as “stepping stones” for species migration. We have further assessed their suitability to establish “Other Effective Area‐Based Conservation Measures” (OECMs), based on their resistance values and environmental conditions. A total of 30 areas were identified, covering 5.37 km^2^ and representing 0.02% of the PAN (Figure 6e). These areas share common characteristics: they are located beyond PA boundaries; are located in focal areas along potential species migration corridors; satisfy the requirement of a certain conservation range (area larger than 0.1 km^2^); exhibit minimal resistance values and favorable ecological suitability within the study area; and are surrounded by human communities while offering ecological, social, cultural, and spiritual values.

### 3.3 Contributions of PAN to the Global Conservation Targets

#### 3.3.1 Contributions of PAN to the GBF 2030 Targets

**FIGURE 7:**
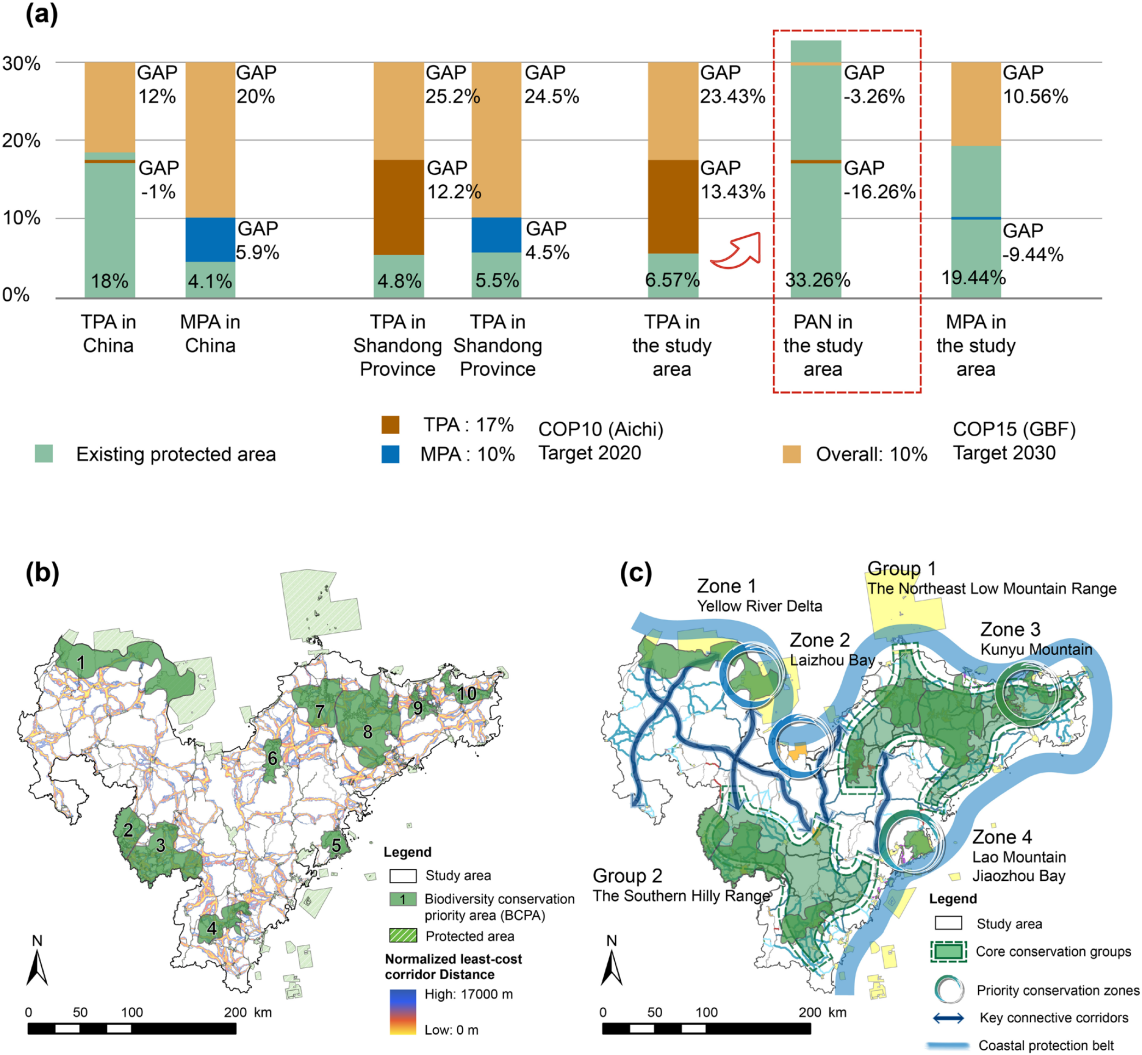
Contributions of the PAN to biodiversity conservation. (a) Area coverage ratios of terrestrial protected areas (TPAs), marine protected areas (MPAs), and the PAN are presented at different scales. The PAN increases PA coverage from 6.57% (existing TPAs) to 33.26%, meeting the GBF's 30 × 30 targets. (b) Shows the overlap between terrestrial BCPAs and the PAN. (c) Proposes an ecological conservation pattern to improve connectivity among PAs along the Shandong Province coast.

When the normalized least‐cost corridor distance is set to 17 k of the average value of the least‐cost distance, the protection of the corridor is extended to 18,660.03 km^2^, accounting for 26.69% of the terrestrial region in the study area. When combined with existing PAs, the coverage increased to 33.26%, surpassing the target of 30% of the area to be protected for biodiversity conservation (Figure 7a). This finding narrows the disparity between the 6.57% PA coverage in Shandong's coastal areas and the GBF's 30 × 30 target. However, it is impractical to strictly protect all identified areas within the PAN of coastal cities on the Shandong Peninsula, as the PAN scope encompasses residential areas and critical transportation infrastructure. Based on analyses of ecological significance and connectivity functions, we identified three major natural land use types with the highest coverage in the PAN as priority conservation targets: forests (11.71% coverage), serving as carbon sinks and habitats for terrestrial protected species; inland waters and wetlands (5.79% coverage), maintaining hydrological balance, filtering urban/agricultural runoff, and supporting endangered migratory birds; and urban greenlands (1.21% coverage) dominated by shrubs and grasses, mitigating the urban heat island effect and enhancing habitat connectivity. In terms of enhancing connectivity for biodiversity conservation, the comparative map reveals that only 20.52% of the BCPAs overlap with PAs (Figure 7b), while counting the extent of the PANs, the overlap rate increases to 54.55%. Higher overlap rates indicated greater conservation effectiveness. Connectivity barriers, particularly between BCPA 5 and 8 and the PAs, were identified (Figure 3f,g). To address these gaps, we proposed an ecological conservation framework comprising a coastal protection belt, two core conservation groups, four priority conservation zones, and multiple key connective corridors (Figure 7c).

#### 3.3.2 Contributions of PAN to the SDGs

**FIGURE 8:**
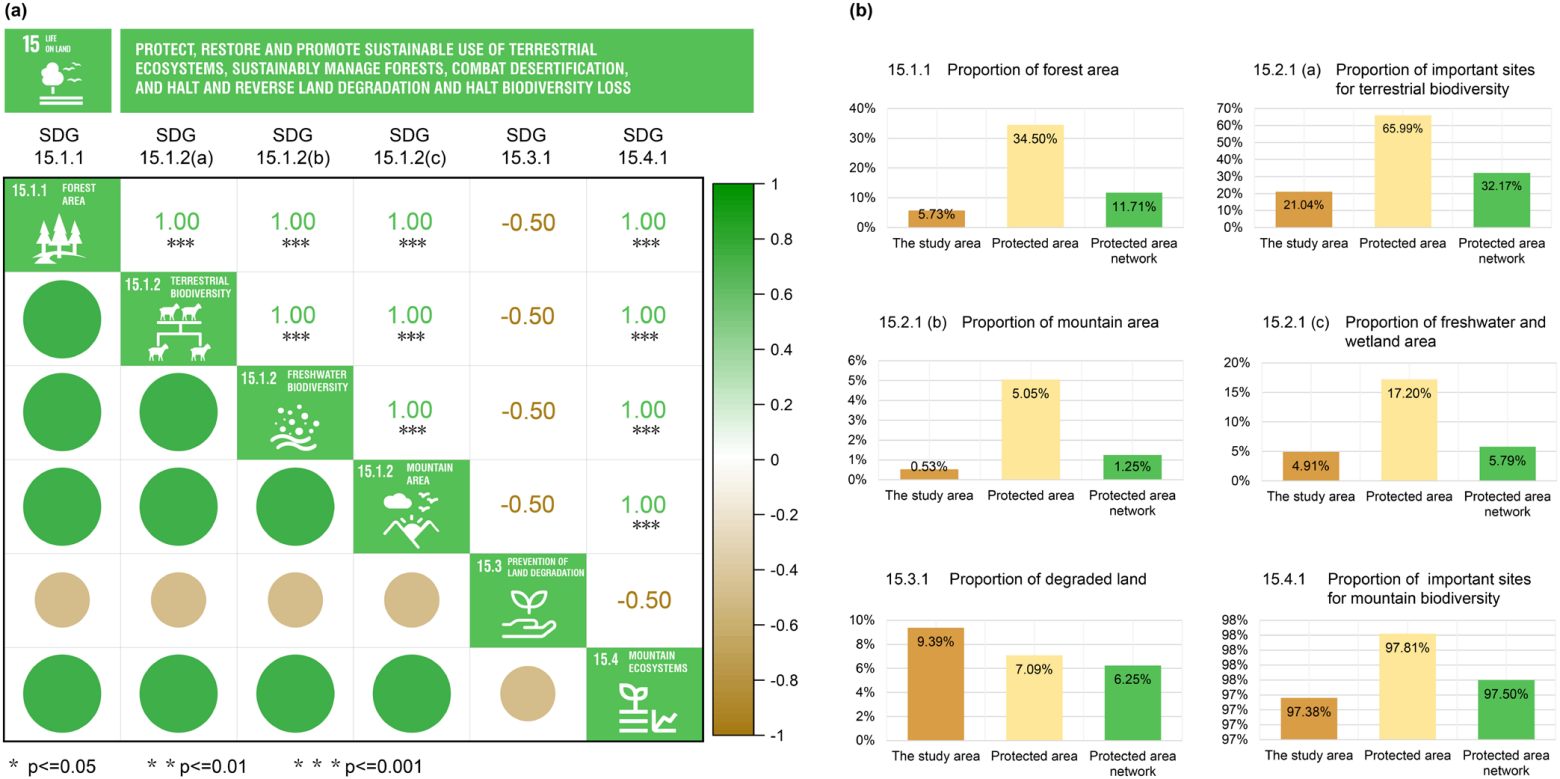
Contributions of the PAN to SDG 15. (a) Spearman correlation coefficient of the SDG 15 indicators. Green circles and coefficients indicate a positive correlation. Yellow circles and coefficients indicate a negative correlation. (b) The capacity of PAs, PAN, and the overall study area to achieve the indicators of SDG 15.

The results indicated that PAs demonstrated the highest capacity to meet the targets of SDG 15.1.1, 15.2.1, and 15.4.1, affirming their priority in protecting, restoring, and promoting the sustainable use of terrestrial ecosystems and curbing biodiversity loss (Figure 8b). The PAN performs intermediately between the PAs and the overall regional levels in nearly all indicators and even outperforms the PAs in terms of the proportion of land degradation, a benefit derived from the deliberate avoidance of areas with high anthropogenic disturbance and high resistance during the PAN construction process. These statistics highlight the comparative advantages of PAN over other territorial areas in achieving SDG 15. The identified spatial profile of PAN can assist local governments in implementing more precise conservation management. Furthermore, by analyzing the achievement of several quantifiable indicators within the SDG 15 sub‐goals, we further examined the intercorrelations among these sub‐goals, finding strong positive correlations among protective indicators and a notable negative correlation between regional protection and regional degradation (Figure 8a). This confirms the interactivity among sub‐goals within the complex framework of the SDGs, suggesting that PANs not only directly enhance quantifiable indicators of sustainable development but also indirectly yield other ecological or social benefits that are difficult to quantify (Appendix E).

## 4 Discussion

### 4.1 Unique Contributions and Potential of PANs in Coastal Regions

Numerous studies have confirmed the significant contributions of coastal PAs to biodiversity conservation, climate change mitigation (Manes et al. 2023), ES provision (Floris et al. 2020), and sustainable development (Rees et al. 2018). However, there is a gap in the detailed discussion on connecting PAs in coastal regions, particularly in densely populated areas. This study proposes a coastal PAN framework that integrates natural and anthropogenic factors to expand and connect existing terrestrial and MPAs. The results of the study show that areas of high resistance values in the coastal zone are directly linked to densely populated town centers and transportation facility areas. The construction of a coastal PAN will facilitate the targeted expansion of PAs and OECMs, mitigate habitat fragmentation, and enhance ecological connectivity, directly contributing to achieving GBF 2030 Targets 2 and 3 (Butchart et al. 2012; Goettsch et al. 2019). Through network modeling, it highlights the importance of the coastal PAN in addressing regional ecological pressures, aligning with previous findings (Lidicker et al. 2019; Saura et al. 2018) that emphasize the critical role of network connectivity in PA management. By linking fragmented habitats, PANs facilitate species migration, reproduction, and dispersal while maintaining regional ecological processes and supporting natural material and energy flows.

Coastal PANs provide specific spatial planning solutions to achieve the GBF 2030 targets. Watson et al. (2023) stressed that PA expansion is crucial for fulfilling national conservation commitments. Wang et al. (2024) explored ecological security patterns in Xishuangbanna to explore spatial planning responses to GBF targets but did not clarify their contributions. This study goes further by showing that coastal PANs extend beyond existing PAs to include natural landscapes, such as mountains, forests, freshwater areas, and coastal zones, which enhance habitat connectivity and serve as potential PA expansion zones (Robinson et al. 2024). These areas are critical for meeting GBF 2030 Targets 2 and 3 and, when integrated with nature‐based solutions and ecosystem‐based approaches, can support other targets (e.g., Targets 4, 8, 11, and 12) (Seddon 2022). Incorporating these actions into urban development and local policies ensures broader alignment with biodiversity goals.

Coastal PANs also significantly contributed to the achievement of the SDGs. Lafortune et al. (2020) emphasized that progress on SDGs requires the combined use of diverse methods and locally adapted indicators; Rees et al. (2018) highlighted advances in marine PANs for achieving Aichi Target 11 and SDG 14 but noted insufficient ecological coherence. This study quantifies coastal PAN contributions to SDG 15 and their direct and indirect impacts on other SDGs, demonstrating that coastal PANs support ES and human well‐being while ensuring socio‐ecological coherence. Overall, these findings underscore the dual role of coastal PANs in ecological conservation and global target achievement.

### 4.2 Countermeasures to Promote the Implementation of Global Conservation Targets

#### 4.2.1 Establish a Comprehensive Evaluation Framework for PANs

The GBF 2030 sets 23 action‐oriented global targets for urgent action from this decade to 2030, and the 2030 Agenda for Sustainable Development encompasses 17 SDGs and 169 specific targets. The quantification of biodiversity‐related indicators is more challenging than that of social and economic indicators (Zhang et al. 2022). Therefore, it is essential to develop a multiparametric assessment framework that incorporates indicators such as biodiversity indices, ES value, climate change mitigation capacity, and socioeconomic synergy to provide a comprehensive evaluation of PAN effectiveness (Chen et al. 2023; Zhao, Zhao, et al. 2024).

#### 4.2.2 Integrate PAN Boundaries Into Territorial Spatial Planning

The “Ecological Conservation Redline” (ECR) was proposed as a conservation concept in China in 2011 and formed a nationwide practical strategy by 2017. Serving as a complement to the scale of PAs, the ECR allows limited human activity within designated zones to balance ecological conservation with social benefits (Zhang et al. 2020). Similarly, OECMs recognized under the Kunming–Montreal GBF can be integrated alongside PAs and ECRs to capture a broader suite of conserved or sustainably managed areas, further strengthening overall network connectivity and conservation outcomes (CBD 2018; Hilty et al. 2020). By aligning PAN boundaries with the ECR and incorporating them into the territorial spatial planning framework, the protective efficacy of PANs can be ensured to gain formal government recognition.

#### 4.2.3 Establish a Multi‐Stakeholder Collaborative Mechanism

The construction of coastal PANs relies on a collaborative mechanism involving multiple stakeholders, including governments, research institutions, communities, and international organizations, owing to the trans‐regional characteristics of species migration and ES (Chu et al. 2022). Such a collaboration is essential for achieving global conservation targets. This mechanism should not only focus on building cross‐regional corridors but also on sharing conservation data and technical expertise (Ortega‐Alvarez et al. 2017). Establishing a collaboration mechanism involving multiple stakeholders, resources, funding, and technical support from various parties can ensure the long‐term and effective management of coastal PANs, thereby advancing the achievement of both the GBF 2030 targets and SDGs on a global scale.

### 4.3 Implications for Constructing and Optimizing a PAN in Coastal Regions

Coastal regions confront significant challenges in constructing networks that connect fragmented habitats due to high levels of anthropogenic disturbance and the ecological vulnerability of coastal ecosystems (Zhao, Qian, et al. 2024). Nevertheless, such efforts are of great strategic importance. They align with China's initiatives to “build ecological corridors and restore important habitats” (General Office of the CPC Central Committee and General Office of the State Council 2019) and fulfill the strategic goal of “improving the spatial network for biodiversity conservation” (Ministry of Ecology and Environment of the People's Republic of China 2024). In addition, the PANs highlight the unique contribution and potential of coastal areas in achieving global conservation targets. Effective implementation of this strategy requires quantifying corridor widths, determining the areas needed for protection, and identifying key zones that significantly influence connectivity (Cao et al. 2020). In this context, this study constructed a PAN with several key advantages. (1) Integration of natural and social influences: the PAN integrates natural and social influences by integrating ecological suitability surfaces with anthropogenic disturbance surfaces, thus reflecting both habitat quality and human impact in coastal regions. (2) Flexible network structure: built using least‐cost modeling and circuit theory, the network generates adaptable boundaries. Structural optimization provides critical decision‐making tools for local governments to develop targeted conservation strategies. (3) Quantification of global contributions: the PAN raises effective PA coverage from 6.57% to 33.26%, surpassing the GBF 30 × 30 target; raises overlap with BCPAs from 20.52% to 54.55%; and improves SDG 15 performance by incorporating 11.71% forest, 5.79% inland water and wetland, and 1.21% urban greenland within its network. By constructing and optimizing an effective coastal PAN, this study aims to enhance ecosystem connectivity and resilience, thereby supporting the attainment of GBF 2030 targets and SDG goals. Additionally, it offers a practical reference model for other coastal regions facing similar ecological and anthropogenic pressures worldwide. The methodology is broadly applicable but should be adjusted to account for local conditions by recalibrating evaluation factors and weights (Lafortune et al. 2020).

### 4.4 Limitations and Future Work

Although PANs constructed based on heterogeneous landscapes have significant advantages (Barnett and Belote 2021; Damschen et al. 2019), there are still improvements to be expected. Kumar and Cushman (2022) highlight that beyond traditional resistance‐based approaches, incorporating spatiotemporal variation, species interactions, and other context‐dependent effects into connectivity models can broaden conservation methodologies and improve their practical effectiveness. To conserve habitat connectivity for specific species, it is essential to collect detailed population distribution and dispersal data for constructing targeted conservation networks. Numerous studies provide valuable references for localized conservation efforts focused on specific species (Diniz et al. 2022; Liu et al. 2025; Santangeli et al. 2023).

Climate change (McKay 2023) and marine ecological conservation (Giakoumi et al. 2018) have become increasingly important in recent years and are explicitly linked to GBF 2030 targets 2, 3, 8, 10, and 11, as well as SDGs 2, 6, 11, 13, and 14. These targets are interconnected, and a well‐designed PAN can enhance regional resilience, mitigate environmental and climate risks (Albert et al. 2017; Xu et al. 2024), and strengthen ecological connectivity between terrestrial and marine ecosystems. Advanced techniques such as population genetics theory (Gagnaire et al. 2015) and hydrodynamic models of larval dispersal (Green et al. 2015) can be employed to model MPA corridors, allowing for dynamic quantification of connectivity patterns in marine areas. By combining the connectivity patterns of terrestrial and marine PANs, this approach provides a holistic framework for coastal conservation. Extending this study to China's entire coastline could evaluate the effectiveness of national‐scale coastal PANs in achieving the GBF 2030 targets and SDGs.

## 5 Conclusions

This study proposes a PAN framework to enhance the structural and functional connectivity in coastal regions and assesses its capacity to contribute to global conservation targets. The main findings are summarized as follows. First, the PAN consists of three PA patch centrality classes, nine linkage pathway categories, eight ecoregions containing pinch and barrier points, and 30 stepping‐stones that provide clear conservation schemes for local governments. Second, when the least‐cost corridor distance matches the average PA patch distance, the PAN area coverage is 33.26%, which exceeds the GBF conservation target of 30%. Third, PAN performs approximately 50% better than the regional averages in achieving coverage‐based indicators for SDG 15 and supports other SDGs through synergistic effects.

Our study demonstrates that coastal PANs can significantly increase coverage and enhance connectivity among PAs, which has a positive effect on bridging the gap between the current scale of protection and the GBF 2030 targets and the SDGs. However, it is essential to recognize that the identification of PANs is insufficient to address biodiversity crises. Effective action focusing on the restoration, protection, and management of areas covered by PAN is the key to addressing regional biodiversity loss and achieving sustainable development.

## Author Contributions

**Ye Zhao:** conceptualization (lead), formal analysis (equal), funding acquisition (lead), methodology (equal), project administration (lead), supervision (lead), validation (equal), writing – original draft (equal). **Xinyu Liu:** data curation (equal), methodology (equal), resources (equal), software (lead), visualization (equal), writing – original draft (equal), writing – review and editing (equal). **Wenyu Zhang:** data curation (equal), methodology (equal), resources (equal), software (equal). **Nan Liu:** data curation (equal), investigation (equal), methodology (equal), resources (equal), software (equal), supervision (equal). **Lin Fan:** data curation (equal), investigation (equal), methodology (equal), resources (equal), software (equal), supervision (equal). **Li Zhao:** data curation (equal), investigation (equal), methodology (equal), resources (equal), software (equal), supervision (equal).

## Conflicts of Interest

The authors declare no conflicts of interest.

## Data Availability

The data that support the findings of this study are openly available in Zenodo at https://doi.org/10.5281/zenodo.15819664.

## Appendix A Area of Various Categories of Protected Areas (PAs) in the Study Area

**Table:**

Region	Protected area categories	Quantity	Area (km^2^)	Area percentage (of the total PA area)
The study area with a total terrestrial area of 69,921.32 km^2^ (terrestrial area) and 8404.00 km^2^ (marine area)	Nature reserve	29	9038.82	69.54
	Scenic spot	17	1035.87	7.97
	Forest park	38	621.70	4.78
	Geological park	8	87.48	0.67
	Wetland park	60	656.25	5.05
	Marine park	19	1557.61	11.99

## Appendix B The Process of Assigning Weights to Resistance Factors and Categorizing Resistance Values

1. Ten experts were invited to participate in the development of resistance factor weights for this study, including the staff of the National Forestry and Grassland Administration (*n* = 2), university professors in this research field (*n* = 3), wildlife conservation enthusiasts (*n* = 2) and local residents (*n* = 3). The experts scored the factors according to the degree of their impact on PA connectivity.

We made a quantitative questionnaire to collect and sort out their opinions.

**Table:**

Type of expert		The staff of the National Forestry and Grassland Administration			University professors			Wildlife conservation enthusiasts		Residents	
Number of persons		2			3			2		3	
Natural suitability factor 1	Elevation, elevation is similar to sea level, the higher the value, the higher the terrain and the greater the impediment to connectivity. To what extent do you think elevation affects PA connectivity? (On a scale of 1 to 10, the higher the score, the greater the degree of influence.)										
	Score	1	2	3	4	5	6	7	8	9	10
	Quantity	1	0	0	1	2	0	1	2	3	0
	Total score: 65					Average score: 6.5					
Natural suitability factor 2	Slope: The slope reflects the steepness of the terrain; the higher the hill, the greater the impediment to connectivity. To what extent do you think slope affects PA connectivity? (On a scale of 1 to 10, the higher the score, the greater the degree of influence.)										
	Score	1	2	3	4	5	6	7	8	9	10
	Quantity	1	0	0	0	1	1	3	1	3	0
	Total score: 68					Average score: 6.8					
Natural suitability factor 3	Relief reflects the terrain relief in a certain area; the higher the degree of the relief, the greater the impediment to connectivity. To what extent do you think relief affects PA connectivity? (On a scale of 1 to 10, the higher the score, the greater the degree of influence)										
	Score	1	2	3	4	5	6	7	8	9	10
	Quantity	1	0	0	1	0	2	4	1	0	1
	Total score: 63					Average score: 6.3					
Natural suitability factor 4	Normalized difference vegetation index (NDVI): vegetation cover creates food sources and habitats for species; the lower the index the greater the impediment to connectivity. To what extent do you think NDVI affects PA connectivity? (on a scale of 1 to 10, the higher the score, the greater the degree of influence)										
	Score	1	2	3	4	5	6	7	8	9	10
	Quantity	1	0	0	0	1	2	2	2	2	0
	Total score: 66					Average score: 6.6					
Natural suitability factor 5	Distance to water bodies, which creates food sources and habitats for species; the greater the distance to water bodies, the greater the impediment to connectivity. To what extent do you think the distance to water affects PA connectivity? (On a scale of 1 to 10, the higher the score, the greater the degree of influence.)										
	Score	1	2	3	4	5	6	7	8	9	10
	Quantity	0	0	1	0	3	1	3	1	1	0
	Total score: 62					Average score: 6.2					
Human interference factor 1	Land use types: The study area contains six types of land use types (urban/township, bare ground, cultivated land, water/wetland, grassland, and forest land). Different land use types affect environmental suitability, and the greater the anthropogenic use of the type the greater the impediment to connectivity. To what extent do you think land use types affect PA connectivity? (on a scale of 1 to 10, the higher the score, the greater the degree of influence)										
	Score	1	2	3	4	5	6	7	8	9	10
	Quantity	0	0	0	0	1	2	1	2	4	0
	Total score: 76					Average score: 7.6					
Human interference factor 2	Distance from roads: the road type in this study contains three categories (expressways, primary roads, and secondary roads). Different road classes and distances have different impacts on the habitat and movement of species, and the closer the road, the greater the impediment to connectivity. To what extent do you think distance from roads affects PA connectivity? (On a scale of 1 to 10, the higher the score, the greater the degree of influence)										
	Score	1	2	3	4	5	6	7	8	9	10
	Quantity	0	0	0	0	4	1	1	1	2	1
	Total score: 69					Average score: 6.9					
Human interference factor 3	Distance from settlements, where human beings gather to live, becomes settlements, and in most cases the denser the settlement and the closer it is to the settlement, the greater the disturbance to biological survival and the greater the impediment to connectivity. To what extent do you think the distance from settlements affects PA connectivity? (on a scale of 1 to 10, the higher the score, the greater the degree of influence)										
	Score	1	2	3	4	5	6	7	8	9	10
	Quantity	0	0	0	0	2	1	3	3	1	0
	Total score: 70					Average score: 7.0					
Synthesize judgment	Summarizing the above judgments, what do you think is the degree of influence of natural environmental factors (elevation, slope, topographic relief, vegetation cover, and distance to water bodies) and human activity factors (type of land use, distance to roads, and distance to settlements) on the connectivity between nature conservation? (1–6 points; the higher the score, the greater the degree of influence)										
	Natural Suitability Factor	2	3	2	3	2	0	4	3	2	4
	Human Interference Factor	4	3	4	3	4	6	2	3	4	2
	The average score for natural suitability factors: 2.5					The average score for human Interference factors: 3.5					

2 In the framework of the Analytic Hierarchy Process (AHP), pairwise comparisons are conducted to generate a decision matrix. Through subsequent calculations, the weights of each factor are derived. To ensure the reliability of these judgments, a Consistency Ratio (CR) is utilized to filter out inconsistent decisions from the pairwise comparison process. Using the *yaahp* software based on AHP principles, experts' evaluations were transformed into weights for each factor (Hurley et al. 2009). The weight of a factor is directly proportional to its assigned score, and the cumulative sum of all factor weights equals 1. This approach allows for a systematic and quantitative assessment of factor importance within the study's context.
3 Table of weights of resistance factors and grading of resistance values.

**Table:**

Number	System‐level	Weight	Index level	Sub‐weight	Classification level	Score
1	Natural suitability factors	0.4167	Elevation/m	0.0836	< 50	1
					50–100	10
					100–300	25
					300–500	50
					> 500	100
2			Slope/(°)	0.0874	> 25	100
					15–25	50
					5–15	25
					2–5	10
					0–2	1
3			Relief	0.0818	> 15	100
					12–38	50
					12–23	25
					5–12	10
					0–5	1
4			Normalized Difference Vegetation Index (NDVI)	0.0849	0–0.18	100
					0.18–0.38	50
					0.38–0.54	25
					0.54–0.72	10
					> 0.72	1
5			Distance to water bodies/m	0.0790	< 500	1
					500–1000	10
					1000–1500	25
					1500–2000	50
					> 2000	100
6	Human interference factors	0.5833	Land use types	0.2062	Urban/township	100
					Bare ground	75
					Cultivated land	50
					Water/wetland	25
					Grassland	10
					Forest land	1
7			Distance from expressways/m	0.0857	> 2000	1
					1500–2000	10
					1000–1500	25
					500–1000	50
					0–500	100
8			Distance from primary roads/m	0.0236	> 1500	1
					1000–1500	10
					1000–500	25
					500–300	50
					0–300	100
9			Distance from secondary roads/m	0.0236	> 1000	1
					500–1000	10
					300–500	25
					100–300	50
					0–100	100
10			Distance from settlements/m	0.1899	> 500	1
					200–500	10
					100–200	25
					50–100	50
					0–50	100

## Appendix C Statistical Results on Indicators for the SDG 15

**Table:**

Indicator	Formula	Percentage (%)	Formula	Percentage (%)	Formula	Percentage (%)
Goal	15.1 By 2020, ensure the conservation, restoration, and sustainable use of terrestrial and inland freshwater ecosystems and their services, in particular forests, wetlands, mountains, and drylands, in line with obligations under international agreements.					
15.1.1 Forest area as a proportion of total land area	Area of forest area in the study area/area of the study area	5.73	Area of forest area in the PAs/area of the PAs	34.50	Area of forest area in the PAN/area of the PAN	11.71
15.1.2 Proportion of important sites for terrestrial and freshwater biodiversity that are covered by protected areas, by ecosystem type	Area of terrestrial biodiversity sites in the study area/area of the study area	21.04	Area of terrestrial biodiversity sites in the PAs/area of the PAs	65.99	Area of terrestrial biodiversity sites in the PAN/area of the PAN	32.17
	Area of mountain area in the study area/area of the study area	0.53	Area of mountain area in the PAs/area of the PAs	5.05	Area of mountain area in the PAN/area of the PAN	1.25
	Area of freshwater and wetlands in the study area/area of the study area	4.91	Area of freshwater and wetlands in the PAs/area of the PAs	17.20	Area of freshwater and wetlands in the PAN/area of the PAN	5.79
Goal	15.3 By 2030, combat desertification, restore degraded land and soil, including land affected by desertification, drought and floods, and strive to achieve a land degradation‐neutral world					
15.3.1 Proportion of land that is degraded over total land area	Area of degraded land in the study area/area of the study area	9.39	Area of degraded land in the PAs/area of the PAs	7.09	Area of degraded land in the PAN/area of the PAN	6.25
Goal	15.4 By 2030, ensure the conservation of mountain ecosystems, including their biodiversity, in order to enhance their capacity to provide benefits that are essential for sustainable development					
15.4.1 Coverage by protected areas of important sites for mountain biodiversity	Area of mountain biodiversity important sites in the study area/area of the study area	97.38	Area of mountain biodiversity important sites in the PAs/area of the PAs	97.81	Area of mountain biodiversity important sites in the PAN/area of the PAN	97.50

*Note:* All data on the area mentioned above are limited to terrestrial and inland freshwater areas, excluding marine regions. Area of terrestrial biodiversity sites in the PAs/area of the PAs (proportion of biodiversity sites within PAs).

## Appendix E

The 2030 Agenda for Sustainable Development emphasizes the role of biodiversity in achieving SDG 14 and 15. Furthermore, studies have demonstrated interdependencies among SDGs, presenting synergies or trade‐offs (Huan and Zhu 2023; Liu et al. 2023), highlighting the direct support of biodiversity toward the realization of the other ten SDGs (1, 2, 3, 6, 7, 8, 9, 11, 12, 13) and indirectly influencing the remaining five SDGs (4, 5, 10, 16, 17) (Blicharska et al. 2019).

The protection of ecosystem diversity by coastal PANs directly supports the other SDGs. For example, forests, beaches, wetlands, and urban parks within coastal cities form a network that strengthens urban resilience and adaptability to climate change, contributing to SDGs 11 and 13 (Jones et al. 2012; Seddon 2022). Mountains and forests within PAN nourish freshwater systems, wetlands, and groundwater, improving water quality and flood resistance, and forming a resilient water cycle system aligned with SDG 6 (Zhou et al. 2015). Environmental improvements reduce air, soil, and water pollution, directly benefit human health and well‐being, and indirectly support SDG 3 (Santos et al. 2017).

The containment of biodiversity loss through coastal PANs also directly supports the other SDGs. The proportion of degraded land within PANs is relatively low, allowing for the effective conservation of soil microorganisms, pollinating flora, and insects, which maintain local genetic resources and impact agricultural productivity (Duffy et al. 2017). This, in turn, helps to eradicate hunger, ensure food security, promote sustainable agriculture (SDG 2), and alleviate poverty (SDG 1).

Furthermore, coastal PANs encompass numerous natural and cultural heritage sites along with surrounding communities that coexist with them. The effective protection of these areas directly contributes to SDG 11 (Zhao, Zhao, et al. 2024). Implementing sustainable tourism policies on this foundation can create employment opportunities and promote local culture and products, indirectly contributing to SDGs 4 and 8 (D'Arco et al. 2021).
